# Critical Evaluation of Assessment and Decision-Making in Complex Limb Fracture Management: A Review of Current Practice and Emerging Trends

**DOI:** 10.7759/cureus.96229

**Published:** 2025-11-06

**Authors:** Kayaththery Varathan, Havil Stephen Alexander Bakka, Adele Zacken, Daniel I Koshy, Jasmin Kirupananthan, Mustafa O Albayati, Mohammed A Bedair, Shanmukha Koppolu, Aleksander Rula, Uzair Khan

**Affiliations:** 1 Surgery, Barts Health NHS Trust, London, GBR; 2 Neurosurgery, Royal Sussex County Hospital, Brighton, GBR; 3 General Surgery, Barking, Havering and Redbridge University Hospitals NHS Trust, London, GBR; 4 Trauma and Orthopaedics, Queen Mary University of London, London, GBR; 5 Trauma and Orthopaedics, Royal London Hospital, London, GBR; 6 General Medicine, St Helier Hospital, London, GBR; 7 Trauma and Orthopaedics, Barts Health NHS Trust, London, GBR; 8 Orthopaedic Surgery, Queen Mary University of London, London, GBR; 9 Trauma and Orthopaedics, Ipswich Hospital, East Suffolk and North Essex NHS Foundation Trust, Ipswich, GBR

**Keywords:** complex limb injury, limb injury, limb-salvage surgery, lower limb amputation, lower limb trauma, mangled limb

## Abstract

Complex limb injury involves severe trauma to the bone, muscle, nerves, and vessels, typically resulting from high-energy incidents such as road traffic collisions, falls, or explosions. Prompt assessment and stabilisation are critical due to the life- and limb-threatening nature of these injuries. Management requires a multidisciplinary approach involving orthopaedic, vascular, and plastic surgeons. The decision between limb salvage and amputation depends on numerous factors, including the mechanism of injury, soft tissue damage, ischaemia time, and patient-specific considerations such as age, comorbidities, and psychological impact. Although several scoring systems (such as the Mangled Extremity Severity Score; Nerve Injury, Ischemia, Soft Tissue Injury, Skeletal Injury, Shock, and Age of Patient; and the Ganga Hospital Open Injury Severity Score) exist to guide clinical decision-making, they have limited predictive value for outcomes and are not universally applicable, particularly in upper limb injuries. Advances such as CT angiography and modern trauma networks have improved assessment, but no single tool reliably dictates management. Systematic reviews and cohort studies show similar long-term physical outcomes between amputation and salvage, though psychological results may favour salvage. Ultimately, decisions must be individualised, integrating clinical judgment, patient values, and multidisciplinary expertise to achieve the best functional and holistic outcomes.

## Introduction and background

Complex limb injury (CLI) can be defined as a subset of traumatic injury which involves severe damage to most or all components of limb architecture, chiefly the osseous, muscular, neurovascular, and dermal elements [1]. Due to the inherent nature of damage, such an injured limb usually has threatened viability, which can lead to significant morbidity and mortality if not dealt with as an emergent condition. Important to note is that in daily practice, the use of the colloquial term “mangled limb” refers to an injury that involves at least three of the aforementioned systems [2], although in local practice, there may be inconsistency with its usage.

As can be expected, these injuries often occur secondary to high-energy trauma. In civilian settings, these include a fall from a large height and high-speed road traffic collisions, while in combat environments, they can include high-powered blasts from improvised explosive devices and high-calibre gun wounds. There is considerable overlap between the causative mechanisms of injury between “major trauma” and “complex limb injury” [3]. Therefore, prompt initial assessment and stabilisation of the patient is undertaken as these injuries can be both limb- and life-threatening, as per the advanced trauma life support (ATLS) protocols [4]. Meticulous management must follow involving a multidisciplinary approach which includes inputs from orthopaedic, plastic, and vascular surgeons [5].

A host of prognostic factors are accounted for when weighing treatment pathway decision-making, with the choice of either limb salvage or amputation. These broadly include patient demographics and peri-incident factors, such as the mechanism of injury, the rapidity of emergency service response (and, therefore, tissue viability status), and limb-specific and general clinical assessment status. Often, limb amputation is the best option when there is a threat to life, inevitably leading to a difficult course for both patients and clinicians [6].

Over the last few decades, there has been rapid development in the pathways of care, from the development and maturation of trauma networks, injury prognostication through various scoring systems, to the development of standards for management [7]. This review aims to explore the assessment, decision-making, and treatment strategies in the management of a CLI.

## Review

Pre-hospital assessment and management

Assessment of a CLI is initiated in the pre-hospital setting, with the primary and secondary surveys based on ATLS principles [4]. Key to management in this phase is the stabilisation inherent to the A to E assessment, with increasing focus on control of massive haemorrhage, which recent literature finds is a greater cause of mortality in major trauma, leading to updated algorithms such as the x-ABC (eXsanguinating hemorrhage, Airway, Breathing, Circulation) and the MARCH (Massive hemorrhage, Airway, Respiration, Circulation, and Hypothermia/Head injury)/PAWS (Pain, Antibiotics, Wounds, and Splinting) algorithms that have been developed by the Committee on Tactical Combat Casualty Care (CoTCCC) and are due to be included in the upcoming ATLS 11th edition [8].

Crucial factors to be included in injury assessment, which can dictate and guide management, include vascular status of the limb, the mechanism of the injury, time to initial presentation and the duration of ischaemia (if relevant), concomitant skeletal injuries, and the pre-hospital care received. During the initial assessment, the question that constantly lingers in the background is whether the limb must be amputated or reconstructed; this decision is usually made through the concomitant and simultaneous use of scoring systems that will be discussed in detail in subsequent sections of this review.

A keystone of patient stabilisation will be controlling and ensuring there are no sources of major haemorrhage, as well as identification of all injuries through a head-to-toe secondary survey. CLIs can be assessed using the “look, feel and move” method often used in orthopaedic assessment, including a thorough neurovascular examination, crucially identifying distal pulses to determine whether the limb is neurovascularly intact. Simple manoeuvres such as bleeding source control and immobilisation can increase the chances of a limb being salvaged. The British Orthopaedic Association Standards for Trauma and Orthopaedics (BOAST) also include guidance for the diagnosis and management of arterial injuries [9] associated with extremity fractures and dislocations. Initial management is advised as the control of haemorrhage under direct pressure/using a tourniquet or compressive dressing, while avoiding the use of blind ligation unless as a means of last resort. When direct pressure is inadequate, pneumatic tourniquets placed at the most distal location proximal to the bleeding can be used [10]. Prolonged compressive devices, although used more frequently earlier, are ill-advised and can lead to significant and avoidable morbidity, especially in arenas of combat. There is continuous ongoing research from a first-response point of view to streamline protocols for when they are to be used to both achieve and maintain haemostasis [11]. In hospital settings, the local major haemorrhage protocol should also be initiated as soon as the risk of life-threatening haemorrhage is identified during the initial x-ABC.

In the case of an absent pulse with deformity of the limb, it should be realigned and reassessed for pulses. At the outset, one should look for clinical signs of vascular injury, which can aid in its identification during initial assessment. Frykberg described hard and soft vascular signs of injury, which provide diagnostic clues to help with decision-making [12]. Hard signs include a palpable thrill, audible bruit, active bleeding, haematoma, and absent distal pulses, all of which signal ischaemia and the need for urgent surgical exploration. Soft signs, on the contrary, are those that do not raise suspicion of direct arterial injury but should lead to increased alertness and eventual causal investigation, and can include hypotension, peripheral neurological deficit, and small haematomas [13]. However, care must be exercised when completely relying on these signs. Romagnoli et al. reviewed 1,910 cases with nearly 60% having hard signs of vascular injury and found that hard signs have limits in arterial injury recognition. Instead, a system identifying ischaemic and haemorrhagic signs was recommended [14].

In cases where clinical signs are not definitive, i.e., with evident soft signs but no hard signs, the Ankle Brachial Pressure Index (APBI), a handheld Doppler, or more extensive imaging modalities such as CT angiography or traditional angiography can and have been utilised [15-18]. Care must always be taken while weighing the necessity of further imaging, cognising the delay that diagnostic imaging can add to an already ischaemic limb and the systemic effects contrast can bring.

The APBI and the Arterial Pressure Index (API) are the two most widely recognised vascular screening tests used in emergent trauma settings. The ABPI is defined as the ratio of the systolic blood pressure (SBP) in the extremity distal to the level of injury to the SBP in the brachial artery of an uninjured upper extremity, whereas the API is defined as the ratio of the Doppler arterial pressure of the injured extremity distal to the level of injury to the Doppler arterial pressure in the uninjured extremity. Calculating either the ABI/API score has replaced conventional arteriography in many settings, with studies showing that an ABI/API threshold value of >0.9 reliably excludes arterial injury, by having a specificity of >90% and a negative predictive value of 98% [19-21]. In older patients and other subgroups with an unclear presentation, a difference of >0.1 between the ABI and the API is usually an indication for a diagnostic imaging study [20].

Adequate analgesia should be ensured in the context of such severe trauma, with the National Institute for Health and Clinical Excellence (NICE) guidelines [22] recommending intravenous morphine and intravenous/intranasal ketamine as first- and second-line drug choices, respectively. Appropriate pain medication allows for a fair assessment if compartment syndrome is suspected [23]. Also important is to ensure a tetanus booster and prophylactic intravenous antibiotics, as frequently open injuries expose the injured to a macrocosm of external infective threats.

The mechanism of injury is arguably the single most important prognostic factor deciding whether the injured limb is amenable to reconstruction, with more distal and more uniformly severed injuries having a better prognosis than those that are more proximal or involve torsional, crushing, or traction/degloving forces [24-26].

The duration of ischaemia can equally contribute not just to the viability of the tissues within the limb but also allows for quantifying the threat to the host individual of systemic insults from reperfusion [27]. It is important to remember that warm ischaemia time should not exceed six hours for the lower limb and eight hours for the upper limb as a general rule [2]. Concomitant skeletal injuries need to be assessed and documented during the secondary survey, and imaging requisitioned to identify and manage accordingly [28]. To prevent ischaemia and reperfusion, femoral to radial or cross-limb shunts can be used [29].

Another important element to remember when managing these conditions is the “second hit” phenomenon, which refers to the subsequent physiological insult that early, definitive surgical intervention can cause, leading to the exacerbation of an otherwise smouldering systemic inflammatory response into one that can lead to multi-organ complications [30-32]. Therefore, preference is given to management using damage control orthopaedics principles, which prioritise external fixation and physiological restitution over definitive fracture management [33]. Nonetheless, definitive fixation is targeted within 36 hours as long as resuscitation is adequate to avoid complications associated with delayed fixation [32].

Finally, an important variable to be considered during the initial assessment is the duration of the pre-hospital phase, as the “golden hour” is often the focus of care, an established concept that arises from the understanding of the trimodal peaks of death within the context of traumatic injury. Briefly, the group within the first peak (which arrives within seconds to minutes) unfortunately succumbs to injury, whereas the second peak (minutes to hours) is amenable to survival with aggressive efforts to resuscitate, and this group is usually the focus of care within the golden hour [34]. It is important to note, however, that there has been a lot of deliberation in existing literature around the importance of time as a standalone metric, particularly concerning the difference in civilian and military settings, as indubitably the differences in the mechanism and type of injury play a major role in influencing outcomes [35]. Evidence on total pre-hospital time remains inconclusive, with suggestions of benefit from increased total pre-hospital time being attributed to increased on-scene time by expert paramedics to stabilise the injured [35]. Of interest is the concept of pre-hospital time having a “trial of life” effect for the subset of patients who arrive with vital signs and have severe blunt injuries [36].

Scoring systems and decision-making for definitive surgical management

Principally, the decision often hinges on whether the injury is isolated or part of a constellation of poly-traumatic injuries, where limb salvage is usually postponed and life is prioritised before the limb [28,37-40].

The decision to salvage a limb or amputate is dependent on multiple factors, including patient factors (age, pre-injury baseline, and wishes), the impact of injury on physiological status, soft tissue injury, and contamination. Given the complexities of these injuries, with many presenting as poly-trauma, there is no set management pathway for these patients. Decisions must be made through a multidisciplinary approach at least involving orthopaedics, vascular, and plastics teams. Patients should ideally be definitively managed within major trauma centres, should the trauma network allow, as they are better equipped and advanced with resources and services [41]. This allows for more attempts to salvage the limb than to amputate.

While the majority of available evidence shows that there are no absolute indications for amputation, the only exception to this would be life-preserving treatment to limit blood loss [42]. The most important deciding factor, however, between the two options is the extent of soft tissue injury. Faris et al. identified that the greater the soft tissue damage, which would be identified on examination of the limb on arrival and in theatre, the higher the risk of a premature amputation [43].

Multiple scoring systems are available, which are based on a gamut of factors. These systems are more commonly used for lower extremity injuries, including the most commonly used Mangled Extremity Severity Score (MESS), and others such as Mangled Extremity Syndrome Index (MESI), Predictive Salvage Index (PSI), Hannover Fracture Scale (HFS), Ganga Hospital Open Injury Severity Score (GHOISS), Limb Salvage Index (LSI) and the Nerve Injury, Ischemia, Soft Tissue Injury, Skeletal Injury, Shock, and Age of Patient score (NISSSA) [44]. Table 1 summarises and describes the prognostic factors used within each system [45]. Importantly, Bosse et al., as part of the landmark Lower Extremity Assessment Project (LEAP), found that initially conceived scoring systems, namely, the MESS, NISSA, MESI, PSI, LSI, and the HFS, were not clinically useful, and the low sensitivity of the indices failed to support their validity as predictors for amputation [46-53].

**Table 1:** Scoring systems for complex limb injuries. Legend: + indicates the inclusion of a variable within the scoring system, and - indicates the absence of a variable. Certain variables have descriptors to imply how they are included within the systems. N indicates that the variable is included as a number, whereas X indicates the variable is an arbitrary categorical variable, which may not be standard across different scoring systems. Table credits: Kayatthery Varathan and Uzair Khan. MESS = Mangled Extremity Severity Score; NISSSA = Nerve Injury, Ischemia, Soft Tissue Injury, Skeletal Injury, Shock, and Age of Patient; MESI = Mangled Extremity Syndrome Index; PSI = Predictive Salvage Index; LSI = Limb Salvage Index; HFS = Hannover Fracture Scale; GHOISS = Ganga Hospital Open Injury Severity Score

	MESS [48]	NISSA [45]	MESI [49]	PSI [50]	LSI [51]	HFS [52]	GHOISS [53]
Age (X)	+	-	+	-	-	-	-
Shock (X)	+	+	+	-	-	-	-
Ischaemia (X)	+	+	-	-	-	+	-
Duration of ischaemia (X)	+	-	-	-	+	+	-
Mechanism of injury (X)	+	-	-	-	-	-	-
Skin injury	-	-	+	-	+	+	+
Soft tissue/Muscle injury (X)	-	+	+	+	+	+	+
Skeletal/Bone injury (X)	-	+	+	+	+	+	+
Arterial injury (X)	-	-	+	+	+	-	-
Nerve injury (X)	-	+	+	-	+	+	+
Deep venous injury (X)	-	-	+	-	+	-	-
Wound contamination (X)	-	-	-	-	-	+	-
Time to operative intervention (X)	-	-	+	+	-	-	-
De-periostation (X)	-	-	-	-	-	+	-
Systematic circulation (X)	-	-	-	-	-	+	-
Injury severity score (N)	-	-	+	-	-	-	-
Comorbidity	-	-	-	-	-	-	+

A recent systematic review by Schiro et al. confirmed these findings, and after studying the most commonly utilised scores (MESS, PSI, LSI, NISSSA), found that they are unable to predict functional outcomes following limb salvage [6]. The NICE guidelines, therefore, recommend that the decision to salvage or amputate should not be made based on an injury severity tool/scoring system [22].

A complex injury in the upper limb can add difficulty to management. It varies in anatomy to the lower limb with increased vascularity and lower muscle mass, with an increased warm ischaemia time [54]. Factors affecting decision-making include costly prostheses that are often not well-suited to patients. Furthermore, existing scoring systems are more suited to the lower limb. Savetsky et al. calculated the MESS score for 76 patients who presented with a mangled upper limb (defined as injury to three or more components), with the results showing a link between the score and the complications and hospital stay [55]. The study then went on to suggest a Mangled Upper Extremity score (MUES), which takes into consideration bony injury, whether a fasciotomy is required, degloving injury, and the area of soft tissue defect. Although this scoring system was not validated as a formal scoring system, it was able to successfully predict complications and hospital stay.

Surgical management: initial and definitive

Patients who present with life-threatening injury have been, in the recent past, managed with damage control orthopaedic (DCO) principles as the standard. A detailed history tracing the evolution of concepts in the treatment of patients sustaining orthopaedic trauma is found in the work by Roberts et al. [56]. Briefly, DCO aims to achieve osseous stabilisation and vascular repair, thereby treating the injury that can initiate haemorrhage and a pathological inflammatory response, while steering away from the “second hit” that came with the “Early Total Care,” emphasising early definitive fixation. However, contention emerged on the timing of definitive surgery and the frequency and “overzealous” use of damage control strategies [57].

Therefore, a recently emerging concept is that of Safe Definitive Surgery (SDS), which has been described as a dynamic synthesis of the earlier two strategies, allowing for the utilisation of either strategy (DCO or ETC) through continuous assessment and re-evaluation of the patients’ physiology and subsequent adaptation of the treatment strategy [58].

Akula et al. conducted a meta-analysis to examine the physical and psychological outcomes of both amputees and limb salvage using validated systems to assess quality of life. Patients who underwent limb salvage had better psychological results [59]. There was, however, no significant difference in the physical outcomes, a finding supported by Busse et al., who identified no difference in functional outcomes seven years post-injury with similar disability rates between the two groups [60]. Although there is no difference in outcomes between the two groups, Naique et al. highlighted the importance of amputations being treated in major trauma centres as there were fewer delays in amputation compared to smaller centres [61]. As discussed by Williams et al. [62] and Harris et al. [63], patients undergoing early amputation (once the decision had been made) experienced a reduced rate of complications, shortened hospital stays, and a lower incidence of postoperative infections.

## Conclusions

This study serves as a succinct and comprehensive review of the assessment and management of CLIs, aimed at serving as the gateway for further reading. We highlight that despite the development of scoring systems and advancements in practice, CLI management remains complex, and multidisciplinary team input is essential when considering management approaches while ensuring good functional, physical, and psychological outcomes for the patients.
